# Correlation between loss of Smad4 and clinical parameters of non-small cell lung cancer: an observational cohort study

**DOI:** 10.1186/s12890-021-01480-z

**Published:** 2021-04-01

**Authors:** Xiangjun Guo, Mengmeng Li, Xin Wang, Yun Pan, Jiashu Li

**Affiliations:** Department of Respiratory and Critical Care Medicine, The First People’s Hospital of Lianyungang City, Lianyungang, Jiangsu China

**Keywords:** Non-small cell lung cancer, NSCLC, Smad4, Diagnosis, Evaluation

## Abstract

**Background:**

SMAD4 has been found to be inactivated to varying degrees in many types of cancer; the purpose of this study was to investigate the correlation between SMAD4 expression in non-small cell lung cancer (NSCLC) and clinical pathological parameters.

**Methods:**

The serum concentration of SMAD4 was measured by enzyme-linked immunosorbent assay and its histological expression was quantified by immunohistochemistry.

**Results:**

The serum concentration of Smad4 in patients with NSCLC was lower than that in benign lung disease patients and healthy individuals (*P* < 0.001) and its concentration was related to the histological classification, pathological differentiation, lymphatic metastasis and clinical stage of NSCLC. The sensitivity and specificity of serum Smad4 were 91.56% and 61.56% for screening NSCLC from healthy individuals and 84.55% and 60.36% for screening NSCLC from patients with benign lung disease. Logistic regression analysis showed that the degree of cell differentiation (*P* < 0.001), lymph node metastasis (*P* < 0.001) and clinical stage of NSCLC (*P* = 0.007) affected the expression of Smad4, and had a strong correlation with the expression of Smad4. The expression of Smad4 in NSCLC tissues was lower than that in normal lung tissues (P = 0.009) and its expression was related to the degree of tissue differentiation, lymph node metastasis and clinical stage (P < 0.05).

**Conclusions:**

The downregulation or deletion of Smad4 is related to the malignant biological behavior of NSCLC and serum Smad4 could be considered as a potential molecular indicator for diagnosis and evaluation of NSCLC.

## Background

In recent years, the morbidity and mortality of tumors have been rising in China, among which lung cancer ranks first. It is estimated that the number of lung cancer cases in China in 2015 was 782,000, accounting for 20.65% of new cancers, and non-small cell lung cancer (NSCLC) accounts for about 85% [1]. In histopathology, NSCLC is divided into squamous cell carcinoma, adenocarcinoma, adenosquamous cell carcinoma, and large cell carcinoma, but lung squamous cell carcinoma (LSCC) and lung adenocarcinoma (LAC) account for the vast majority [2]. It has now been recognized that lung cancer is a disease caused by the interaction of external and internal causes. External factors can induce malignant transformation of cells and irreversible gene changes, including the activation of proto-oncogenes, the inactivation of tumor suppressor genes, the activation of self-feedback secretory loops, and the inhibition of apoptosis, resulting in uncontrolled cell growth [3]. These genetic changes are randomly generated in multiple steps over a long period of time. Many genetic abnormalities and the underlying mechanism of lung cancer are unclear, but these changes ultimately involve the loss of control of key physiological functions of cells, including proliferation, apoptosis, differentiation, signal transmission, and migration [3, 4].

In 1996, a deleted in pancreatic carcinoma locus 4 (DPC4) on chromosome 18q21.1 was cloned from pancreatic cancer. The DPC4 gene is composed of 2680 bases and it contains 11 exons and 10 introns. The DPC4 gene encodes a protein consisting of 552 amino acids, which is the Smad4 protein [5]. Recent studies have shown that the mutation, loss and down-regulation of the DPC4 gene are intrinsically linked to some malignant tumors and have played an important role in the development and metastasis of cancer [6–8]. So far, some studies have continuously suggested that there is a complex relationship between lung cancer and DPC4/Smad4 [9, 10], but the research data on the expression of Smad4 in NSCLC and its clinical significance is still insufficient. This study examined the expression of Smad4 in the serum and tissues of patients with NSCLC and analyzeed the correlation between Smad4 and various clinical parameters of NSCLC to understand the role of Smad4 in the diagnosis, risk prediction and disease evaluation of NSCLC.

## Methods

### Involved individuals

**Fig. 1:**
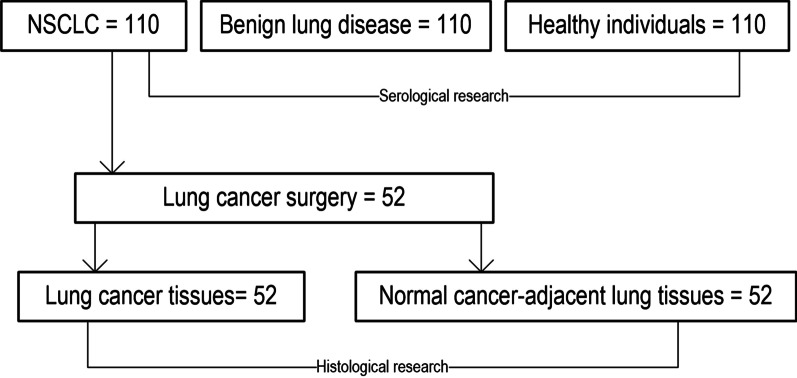
Flow chart for grouping of research objects. NSCLC, non-small cell lung cancer

This study included 110 patients with NSCLC, 110 patients with benign lung disease and 110 healthy individuals that determined by physical examination (The First People’s Hospital of Lianyungang City, Lianyungang, Jiangsu, China) (Table 1). The enrolled individuals in the above three groups were all took peripheral blood samples for serological test. Of the 110 NSCLC patients, 52 received lung cancer resection, and lung cancer tissues and normal cancer-adjacent lung tissues were collected for histological test (Table 2). The path and grouping of patients included are shown in Fig. 1. This project was approved by the Institutional Review Board (The First People’s Hospital of Lianyungang City, Lianyungang, Jiangsu, China), and written informed consent was obtained from all subjects.

**Table 1:** Clinico-pathological features of patients involved in the study

Items	Lung cancer (N = 110)	Benign lung disease (N = 110)	Healthy individuals (N = 110)
Gender			
Male	51 (46.3%)	64 (58.2%)	63 (57.2%)
Female	59 (53.7%)	46 (41.8%)	47 (42.8%)
Ages			
≤ 60	57 (51.8%)	50 (45.5%)	51 (46.4%)
> 60	53 (48.2%)	60 (54.5%)	59 (53.6%)
Smoking			
No	63 (57.2%)	71 (64.5%)	70 (63.6%)
Yes	47 (48.8%)	39 (35.5%)	40 (36.4%)
Histology			
LSCC	62 (56.4%)		
LAC	48 (43.6%)		
Differentiation degree			
Poorly differentiated	34 (30.9%)		
Moderately differentiated	42 (38.2%)		
Well differentiated	34 (30.9%)		
Lymphatic metastasis			
N0–N1	37 (33.7%)		
N2–N3	73 (66.3%)		
Clinical staging			
IA–IIB	52 (47.2%)		
IIIA–IV	58 (52.8%)		

*N* number of patients, *LAC* lung adenocarcinoma, *LSCC* lung squamous cell carcinoma

**Table 2:** Clinical characteristics of patients undergoing lung cancer resection (N = 52)

Items	Lung cancer patients for tissues test	
	Characteristics	Lung cancer patients
Gender	Male	31 (59.6%)
	Female	21 (40.4%)
Ages	< 60	29 (55.8%)
	≥ 60	23 (44.2%)
Smoking	Yes	27 (51.9%)
	No	25 (48.1%)
Histology	LAC	19 (36.5%)
	LSCC	33 (63.5%)
Differentiation degree	Poorly	11 (21.1%)
	Moderately	19 (36.5%)
	Well	22 (42.4%)
Lymphatic metastasis	N0–N1	37 (71.1%)
	N2–N3	15 (28.9%)
Clinical TNM staging	IB–IIB	29 (55.8%)
	IIIA	23 (44.2%)

*LAC* lung adenocarcinoma, *LSCC* lung squamous cell carcinoma, *N* the grade of lymphatic invasion, *TNM* the TNM Classification of Malignant Tumours

### Inclusion criteria for included individuals

Inclusion criteria for patients with lung cancer: (1) must be diagnosed with NSCLC by pathology and reported a clear degree of cell differentiation; (2) blood tests, liver and kidney functions and electrolyte tests were not significantly abnormal; (3) no anti-cancer treatment measures such as chemotherapy, radiotherapy, molecular targeted therapy, etc. were received within 3 weeks prior to specimen collection. Inclusion criteria for patients with benign lung disease: (1) benign pulmonary nodules, organizing pneumonia, old pulmonary tuberculosis, pulmonary interstitial fibrosis, and stable period of chronic obstructive pulmonary disease; (2) not accompanied by lung infection; (3) blood tests, liver and kidney functions and electrolyte tests were not significantly abnormal. Inclusion criteria for healthy people: no history of disease, no abnormalities in blood cell analysis, liver and kidney function, electrolytes, abdominal ultrasound and thyroid ultrasound examination, and chest CT.

### Preparation of blood specimens

The included NSCLC patients, benign lung disease patients and healthy control individuals were grouped according to the pre-designed inclusion order (in chronological order). In the early morning of the second day of enrollment, 5 mL of fasting peripheral venous blood of the enrolled individuals was collected. The collected blood was allowed to stand at room temperature for 30 min and was centrifuged at 3000 r/min for 10 min. The serum on top of centrifuge tube was collected and stored at − 80 °C until testing.

### Tissue microarray (TMA) construction

According to HE staining, the representative spots in the paraffin specimens were marked. Some small holes with a diameter of 1.5 mM were punched on the wax block of the receptor using a fine needle of a tissue array instrument in a pre-designed arrangement sequence. The corresponding portions marked on the donor wax block were drilled to collect tissue cores with a diameter of 1.5 mM. The tissue core was transferred to the hole of the receiver wax block, and the distance between the tissue cores was 0.2 mM. The constructed tissue chip wax block was placed in a 55 °C incubator for 10 min. When the wax of the acceptor wax block was dissolved with the newly inserted cylindrical tissue core, the wax block was removed and stored in a refrigerator at 4 °C for later use. The tissue chip wax block was cut into 4 μm thick tissue slices in a frozen microtome, and the cut tissue pieces were pasted on a glass slide soaked with APES slice adhesive at 58 °C for 18 h, and stored at − 20 °C until use.

### Enzyme-linked immunosorbent assay (ELISA)

The concentration of serum Smad4 was measured by sandwich-type ELISA according to the method provided by the kit (Beijing Andy Huatai Biological Technology Co., Ltd., Beijing, China). The operation steps were as follows. (1) The kit was equilibrated at room temperature for 30 min. (2) The microplate was set as standard and sample wells respectively. (3) 50μL of standards of different concentrations were added to standard wells and 50μL of samples to be tested were added to sample wells, and no samples were added to blank wells. (4) Except for blank wells, 100 μL of Smad4 antibody labeled with horseradish peroxidase (HRP) was added to each of the standard wells and the sample wells. (5) The reaction wells were sealed with a plate sealing membrane and were incubated for 60 min in a 37 °C water bath box. (6) After discarding the liquid in the wells, 350 μL of washing solution were added to each well and were allowed to stand for 1 min, repeating 5 times. (7) 50 μL of substrates A and B were added to each well and were incubated at 37 °C for 15 min in the dark. (8) 50 μL of the stop solution were added to each well, and the optical density value of each well was measured at a wavelength of 450 nm within 15 min.

### Immunohistochemistry (IHC)

The tissue chips were stained using standard biotin–streptavidin–peroxidase method according to the introduction of kit (Bostere Biotech Company, Wuhan, China). The specific method was as follows: (1) The chips were baked in a 60 °C oven for 60 min, dewaxed and hydrated with xylene, and subjected to antigen heat repair with 0.01 M citrate buffer (pH 6.0). (2) After cooling at room temperature for 20 min, the tissue chips were treated with 0.3% Trixon-X100 for 30 min, blocked with 3% hydrogen peroxide-methanol and serum one by one. (3) Smad4 (1:1000, Bostere Biotech Company, Wuhan, China) antibody was added on the tissue chips as a primary antibody, and was incubated at 4 °C overnight. (4) After washing, biotin-labeled secondary antibody was added on the chips and was incubated for 30 min at room temperature. (5) The working solution of horseradish enzyme-labeled streptavidin was added to the chips and was incubated at room temperature for 30 min. (6) DAB was added for color development, and hematoxylin was slightly counterstained. (7) The tissue chips were dehydrated with gradient alcohol and were made transparent with xylene, and were sealed with neutral glue. The Smad4′s immunohistochemical staining were evaluated by a semi-quantitative analysis standard: no tumor cell staining was scored as 0; light staining was scored as 1; moderate staining was scored as 2; more than 75% of tumor cells staining was scored as 3 points; more than 1 point were defined as positive expression. The collection of immunohistochemical images in this study was done using a microscope image analysis system (BD-2000C, Beijing Qihang Boda Technology Co., Ltd. China).

### Statistical analysis

There were two types of data generated in this study: measurement data and count data. The distribution state of the measurement data was determined by the homogeneity test of variance. The statistical analysis of measurement data was completed by Student's t test and analysis of variance. The diagnostic efficacy of the detection index was determined by the receiver operating characteristic (ROC) curve. The correlation between the detection index and the clinical parameters of lung cancer was evaluated by logistic regression analysis. The statistical analysis of count data was performed by chi-square test and Sparman rank correlation test. *P* < 0.05 was considered statistically significant.

## Results

### Serum concentration of Smad4 in NSCLC patients is lower than that in patients with benign lung disease and healthy individuals

**Fig. 2:**
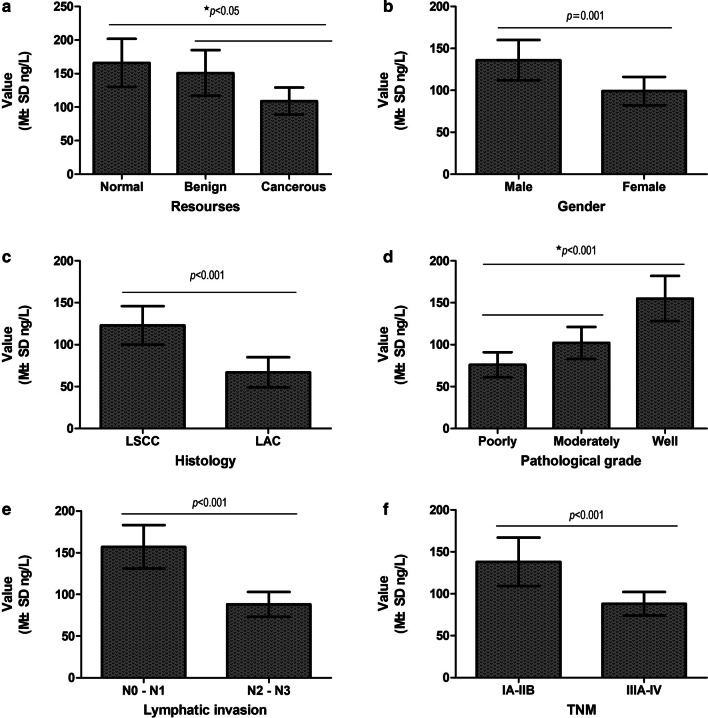
Correlation between clinical features and serum concentration of Smad4 in NSCLC patients. **a** Serum concentration of Smad4 was lower in NSCLC patients than in patients with benign lung disease and healthy people (*P* < 0.05). **b** Serum concentration of Smad4 was lower in female patients than in male patients (*P* = 0.001). **c** Serum concentration of Smad4 was lower in LAC patients than in LSCC patients (*P* < 0.001). **d** Serum concentration of Smad4 was lower in poorly differentiated NSCLC than in moderately and well differentiated NSCLC (*P* < 0.001). **e** NSCLC patients with lymph node metastasis (N2–N3) showed a lower concentration of Smad4 than patients with lymph node metastasis (N0–N1) (*P* < 0.001). **f** NSCLC patients at stages IIIA–IV showed a lower concentration of Smad4 than patients at stages IA–IIB (*P* < 0.001). NSCLC, non-small cell lung cancer; LAC, lung adenocarcinoma. LSCC, lung squamous cell carcinoma. N, node stage (TNM classification)

The concentration of serum Smad4 in patients with NSCLC (109.41 ± 19.51 ng/L) was lower than that in patients with benign lung disease (151.66 ± 34.79 ng/L) and healthy individuals (166.67 ± 36.16 ng/L) (*P* < 0.001) (Table 2; Fig. 2a). However, the concentration of serum Smad4 between patients with benign lung disease and healthy individuals did not show a difference (*P* > 0.05) (Table 3; Fig. 2a).

**Table 3:** Correlation between serum Smad4 concentration and clinico-pathological parameters of NSCLC (N = 110)

Parameter	Group	Concentration of Smad4 in serum of NSCLC						
		Value (ng/L)	Degree of freedom	Test of Homogeneity of Variances		95% CI for Mean	ANOVA	
				Statistical value	*P* value		Statistical value	*P* value
Specimen source	Normal	166.67 ± 36.16	343	2.858	0.075	156.69–177.17	23.698	< 0.001
	Benign	151.66 ± 34.79				139.06–163.63		
	Cancerous	109.41 ± 19.51^a^				96.53–122.12		
Gender	Male	136.34 ± 24.37	108	0.223	0.638	116.58–157.86	3.351	0.001
	Female	99.21 ± 17.03				72.41–109.38		
Ages	< 60	112.27 ± 16.47	108	0.358	0.127	98.68–128.39	1.367	0.313
	≥ 60	107.56 ± 21.88				94.08–112.77		
Smoking	No	118.43 ± 21.09	108	0.692	0.407	98.27–136.55	1.050	0.296
	Yes	102.36 ± 19.17				85.32–122.37		
Histology	LSCC	123.59 ± 23.81	108	3.704	0.057	120.57–152.87	5.171	< 0.001
	LAC	87 ± 18.62				65.21–94.24		
Pathological grade	Poorly	78.65 ± 15.08^b^	107	2.756	0.062	66.21–92.12	21.19	< 0.001
	Moderately	102.47 ± 19.18				78.36–117.89		
	Well	155.82 ± 27.63				127.12–178.23		
Lymphatic invasion	N0–N1	157.67 ± 26.65	108	0.107	0.744	126.41–177.89	5.057	< 0.001
	N2–N3	88.24 ± 15.28				53.49–78.34		
TNM	IA–IIB	138.43 ± 29.10	108	0.156	0.694	120.15–159.03	3.668	< 0.001
	IIIA-IV	88.78 ± 13.22				71.33–107.97		

M ± SD mean ± standard deviation, *ANOVA* analysis of variance, *LAC* lung adenocarcinoma, *LSCC* lung squamous cell carcinoma, *TNM* the TNM Classification of Malignant Tumours

^a^*P* < 0.05, cancerous compared with benign and normal

^b^*P* < 0.05, Poorly compared with moderately and well

### Serum concentration of Smad4 is related to histological classification, pathological differentiation, lymphatic metastasis and clinical stage of NSCLC

The concentration of serum Smad4 had nothing to do with the age and smoking status of NSCLC patients (*P* > 0.05) (Table 3), but seemed to be related to the gender of the patient (*P* = 0.001) (Table 3; Fig. 2b). Smad4 in the serum of LAC patients (87 ± 18.62 ng/L) was lower than that of LSCC (123.59 ± 23.81 ng/L) (*P* < 0.001) (Table 3; Fig. 2c). The serum concentration of Smad4 in poorly differentiated NSCLC (78.65± 15.08 ng/L) was in turn lower than that in moderately differentiated (102.47 ± 19.18 ng/L) and well differentiated NSCLC (155.82 ± 27.63 ng/L) (*P* < 0.001) (Table 3; Fig. 2d). The serum concentration of Smad4 was negatively correlated with the degree of lymph node metastasis of NSCLC (N0–N1: 157.67 ± 26.65 ng/L; N2–N3: 88.24 ± 15.28 ng/L) (*P* < 0.001) (Table 3; Fig. 2e). NSCLC with clinical stage of IIIA–IV (88.78 ± 13.22 ng/L) showed a lower Smad4 serum concentration than NSCLC with clinical stage IA–IIB (138.43 ± 29.10 ng/L) (*P* < 0.001) (Table 3; Fig. 2f).

### Quantitative analysis of serum Smad4 detection for identifying NSCLC

**Fig. 3:**
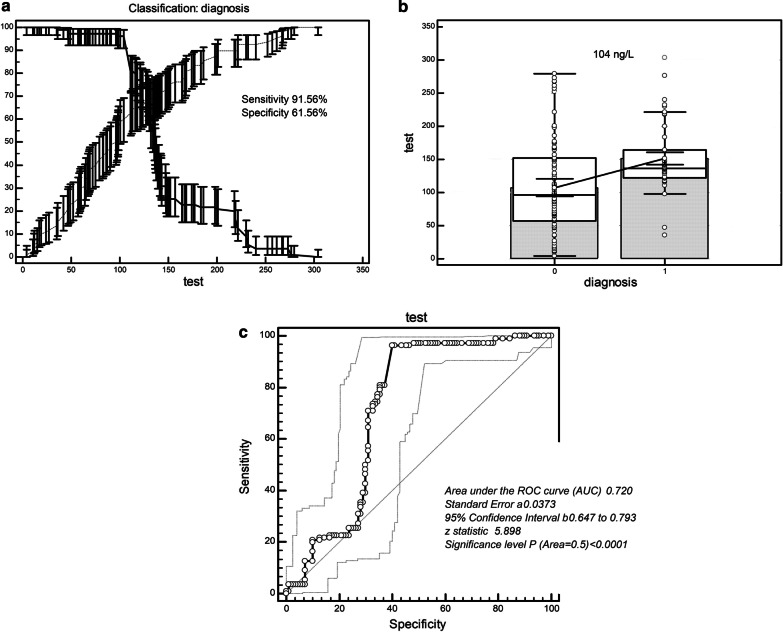
ROC curve analysis of serum Smad4 to distinguish NSCLC patients from healthy people. **a**, **b** Critical value (104 ng/L) of serum Smad4 for discerning NSCLC patients from healthy people had a sensitivity of 91.56% and specificity of 61.56%. **c** ROC of serum Smad4 for distinguishing NSCLC patients from healthy people (AUC = 0.720). NSCLC, non-small cell lung cancer; ROC, receiver operating characteristic curve; AUC, area under the curve

**Fig. 4:**
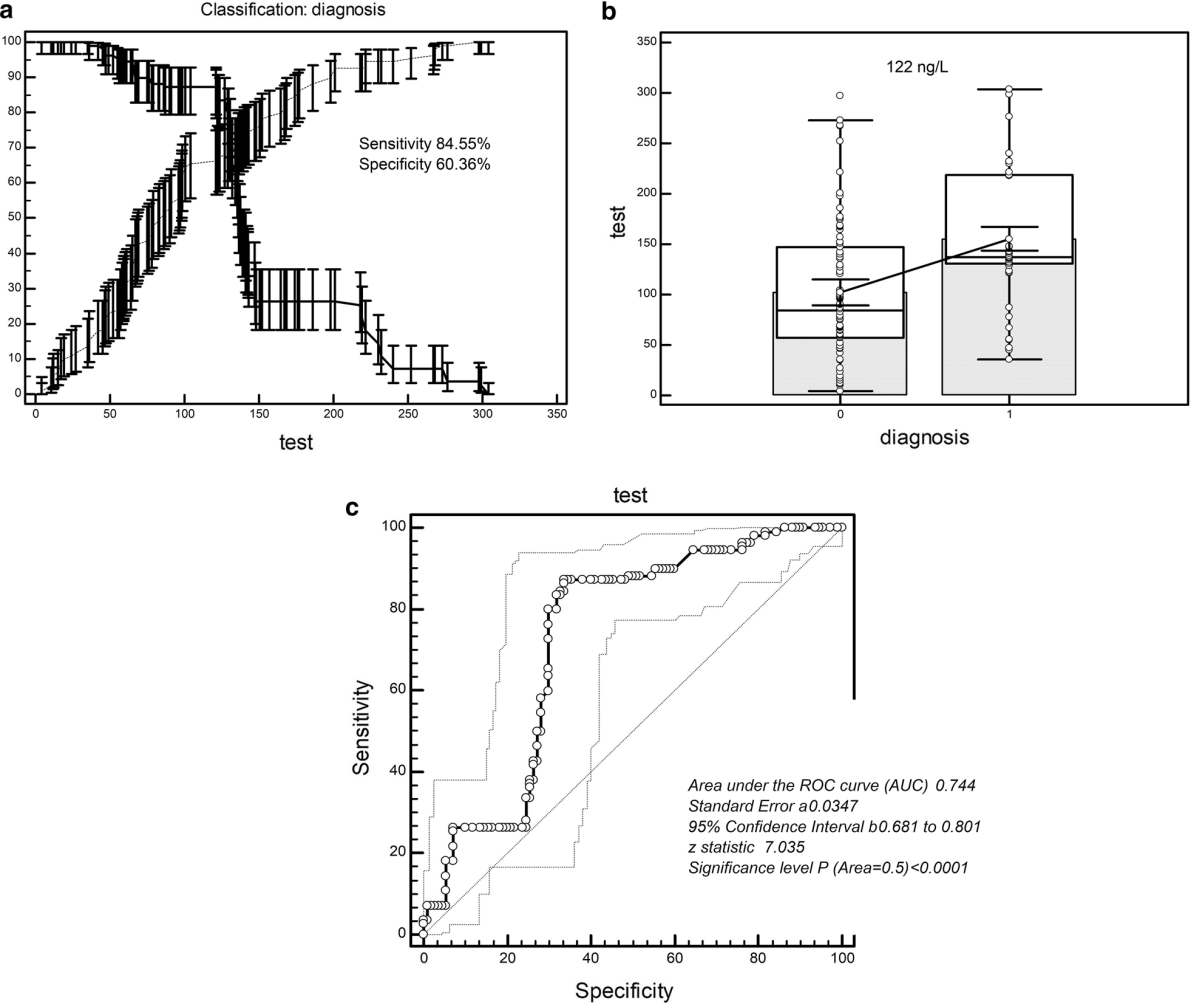
ROC curve analysis of serum Smad4 to distinguish NSCLC patients from benign lung disease patients. **a**, **b** Critical value (122 ng/L) of serum Smad4 for discerning NSCLC patients from benign lung disease patients had a sensitivity of 84.55% and specificity of 60.36%. **c** ROC of serum Smad4 for distinguishing lung cancer patients from benign lung disease patients (AUC = 0.744). NSCLC, non-small cell lung cancer; ROC, receiver operating characteristic curve; AUC, area under the curve

The ROC analysis showed that the diagnostic threshold was 104 ng/L when the serum concentration of Smad4 was used to screen NSCLC from healthy individuals. The sensitivity and specificity of this value were 91.56% and 61.56% (Table 4; Fig. 3a–c). The area under the ROC curve (AUC) was 0.720 (SE = 0.0373; 95% Confidence Interval = 0.647 to 0.793; z statistic = 5.898; *P* < 0.0001). When the serum concentration of Smad4 was used to screen NSCLC from patients with benign lung disease, the diagnostic threshold was 122 ng/L. The sensitivity and specificity of this value were 84.55% and 60.36% (Table 4; Fig. 4a–c). The area under the ROC curve (AUC) was 0.744 (SE = 0.0347, 95% Confidence Interval = 0.681 to 0.801; z statistic = 7.035; *P < *0.0001).

**Table 4:** ROC curve analysis of serum Smad4 distinguishing lung cancer

Criterion	Sensitivity	95% CI	Specificity	95% CI	+ LR	-LR
Calculation of critical value of serum Smad4 in lung cancer and healthy people						
> 35	100.00	96.7–100.0	13.64	7.8–21.5	1.16	0.00
> 46.6	99.09	95.0–100.0	20.91	13.7–29.7	1.25	0.043
> 47	97.27	92.2–99.4	21.82	14.5–30.7	1.24	0.12
> 97	97.27	92.2–99.4	51.82	42.1–61.4	2.02	0.053
> 98	96.36	91.0–99.0	53.64	43.9–63.2	2.08	0.068
> 104*	91.56	81.0–99.0	69.16	50.2–69.2	2.41	0.061
> 123.4	73.64	64.4–81.6	72.36	56.7–78.1	2.19	0.40
> 147.8	25.45	17.6–34.6	79.73	63.4–85.8	0.93	1.02
> 183.4	21.82	14.5–30.7	83.64	75.4–90.0	1.33	0.93
> 239.8	3.64	1.0–9.0	92.73	86.2–96.8	0.50	1.04
> 279.33	0.91	0.02–5.0	100.00	96.7–100.0		0.99
Calculation of the critical value of serum Smad4 in lung cancer and benign lung diseases						
> 35	100.00	96.7–100.0	13.64	7.8–21.5	1.16	0.00
> 56	94.55	88.5–98.0	23.64	16.1–32.7	1.24	0.23
> 65	94.55	88.5–98.0	35.45	26.6–45.1	1.46	0.15
> 86	88.18	80.6–93.6	50.91	41.2–60.6	1.80	0.23
> 122*	84.55	76.4–90.7	60.36	56.7–75.1	2.51	0.23
> 127	83.64	75.4–90.0	62.18	58.6–76.7	2.63	0.24
> 136	58.18	48.4–67.5	71.82	62.4–80.0	2.06	0.58
> 147	33.64	24.9–43.3	75.45	66.3–83.2	1.37	0.88
> 201	26.36	18.4–35.6	92.73	86.2–96.8	3.62	0.79
> 273	7.27	3.2–13.8	99.09	95.0–100.0	8.00	0.94
> 297.4	3.64	1.0–9.0	100.00	96.7–100.0		0.96

*ROC* receiver operating characteristic curve, *95% CI* 95% confidence, + *LR* positive likelihood ratio, − *LR* negative likelihood ratio

^*^Cutoff values to differentiate NSCLC from healthy people and benign lung disease

### Estimation of clinical parameters affecting serum concentration of Smad4

Using the threshold of Smad4 serum concentration as the cut-off point, the serum level of Smad4 was converted into binary variables: positive and negative. Logistic regression analysis was employed to analyze clinical factors that may affect the serum concentration of Smad4. A total of 7 variables, including gender, age, smoking, histology, differentiation, lymphatic invasion, and TNM stage were included as dependent variables (Table 5). The results showed that the degree of cell differentiation (*P* < 0.001), lymph node metastasis (*P* < 0.001) and clinical stage of NSCLC (*P* = 0.007) directly affected the expression of Smad4, and the three showed a strong correlation with the expression of Smad4.

**Table 5:** Logistc regression between serum Smad4 concentration and clinical parameters of NSCLC

Variables in the equation									
Backward deletion	Variables	Regression coefficients	S.E	Wald	df	*p* value	OR value	95% C.I.for EXP (B)	
								Lower	Upper
Step 1^a^	Gender	−.604	.711	.722	1	.396	.546	.135	2.204
	Age	.030	.029	1.078	1	.299	1.031	.974	1.091
	Smoking	.213	.584	.133	1	.715	1.238	.394	3.885
	Histology	2.525	.806	9.818	1	.002	12.485	2.574	60.564
	Differentiation	1.363	.401	11.530	1	.001	3.907	1.779	8.580
	Lymphatic invasion	−.274	.757	.131	1	.717	.760	.172	3.351
	TNM stage	− 1.414	.767	3.400	1	.065	.243	.054	1.093
	Constant	− 4.330	2.096	4.268	1	.039	.013		
Step 2^a^	Gender	−.588	.710	.687	1	.407	.555	.138	2.233
	Age	.029	.029	1.029	1	.310	1.030	.973	1.089
	Smoking	.172	.569	.091	1	.763	1.188	.389	3.624
	Differentiation	2.514	.805	9.763	1	.002	12.360	2.553	59.842
	Lymphatic invasion	1.394	.393	12.579	1	.000	4.032	1.866	8.712
	TNM stage	− 1.605	.558	8.270	1	.004	.201	.067	.600
	Constant	− 4.218	2.059	4.198	1	.040	.015		
Step 3^a^	Gender	−.542	.689	.619	1	.431	.582	.151	2.244
	Age	.028	.028	.954	1	.329	1.028	.972	1.087
	Differentiation	2.491	.796	9.790	1	.002	12.073	2.536	57.471
	Lymphatic invasion	1.364	.376	13.117	1	.000	3.910	1.869	8.178
	TNM stage	− 1.608	.558	8.300	1	.004	.200	.067	.598
	Constant	− 4.005	1.933	4.291	1	.038	.018		
Step 4^a^	Age	.033	.028	1.361	1	.243	1.033	.978	1.091
	Differentiation	2.095	.601	12.131	1	.000	8.122	2.499	26.399
	Lymphatic invasion	1.363	.372	13.400	1	.000	3.908	1.884	8.107
	TNM stage	− 1.542	.546	7.966	1	.005	.214	.073	.624
	Constant	− 4.381	1.895	5.344	1	.021	.013		
Step 5^a^	Differentiation	2.188	.592	13.638	1	.000	8.914	2.791	28.465
	Lymphatic invasion	1.380	.372	13.754	1	.000	3.977	1.917	8.249
	TNM stage	− 1.375	.512	7.203	1	.007	.253	.093	.690
	Constant**	− 2.760	1.202	5.274	1	.022	.063		

*NSCLC* non-small cell lung carcinomas, *S.E.* standard error of regression coefficient, *Wald* test statistics for regression coefficients, *Df* degrees of freedom, *OR* odds ratio, *95% C.I.* 95% confidence interval, *TNM* the TNM Classification of Malignant Tumours

^a^Variable (s) entered on step 1: gender, age, smoking, histology, differentiation, lymphatic invasion and TNM stage; **, Logistc Regression Equation: P = 1/[1 + e^− (−2.760+2.188 differentiation+1.380 lymphatic invasion−1.375 TNM stage)^]

### Expression of Smad4 in NSCLC tissues is lower than that in normal cancer-adjacent lung tissues

**Fig. 5:**
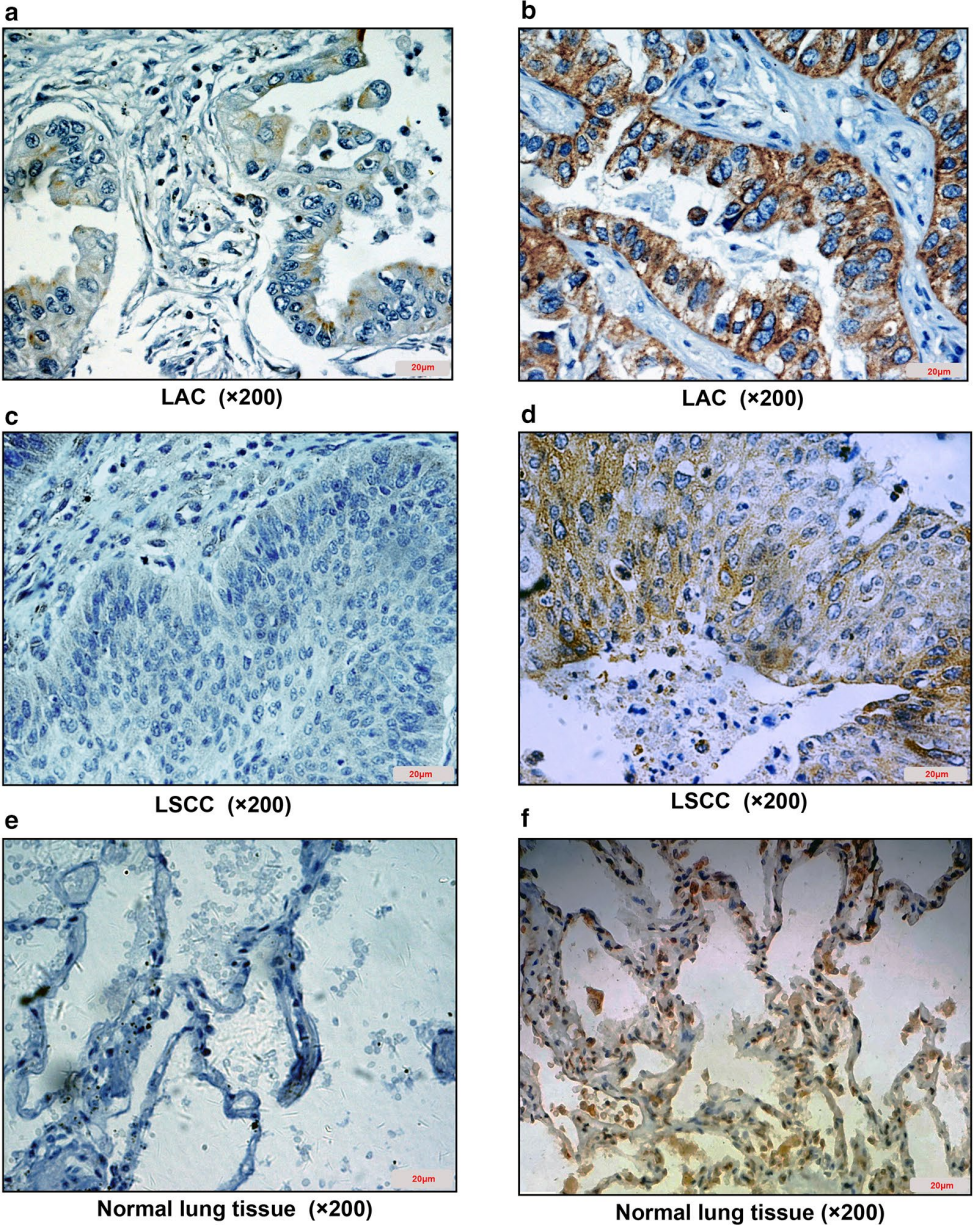
IHC analysis of Smad4 in NSCLC tissues and normal lung tissues adjacent to cancer (IHC × 200). **a** Negative expression of Smad4 in poorly differentiated LAC; **b** Positive expression of Smad4 in well differentiated LAC; (C) Negative expression of Smad4 in poorly differentiated LSCC; **d** Positive expression of Smad4 in well differentiated LSCC; **e** Negative expression of Smad4 in normal lung tissue adjacent to cancer; **f** Positive expression of Smad4 in normal lung tissue adjacent to cancer. NSCLC, non-small cell lung cancer; LAC, lung adenocarcinoma. LSCC, lung squamous cell carcinoma

**Fig. 6:**
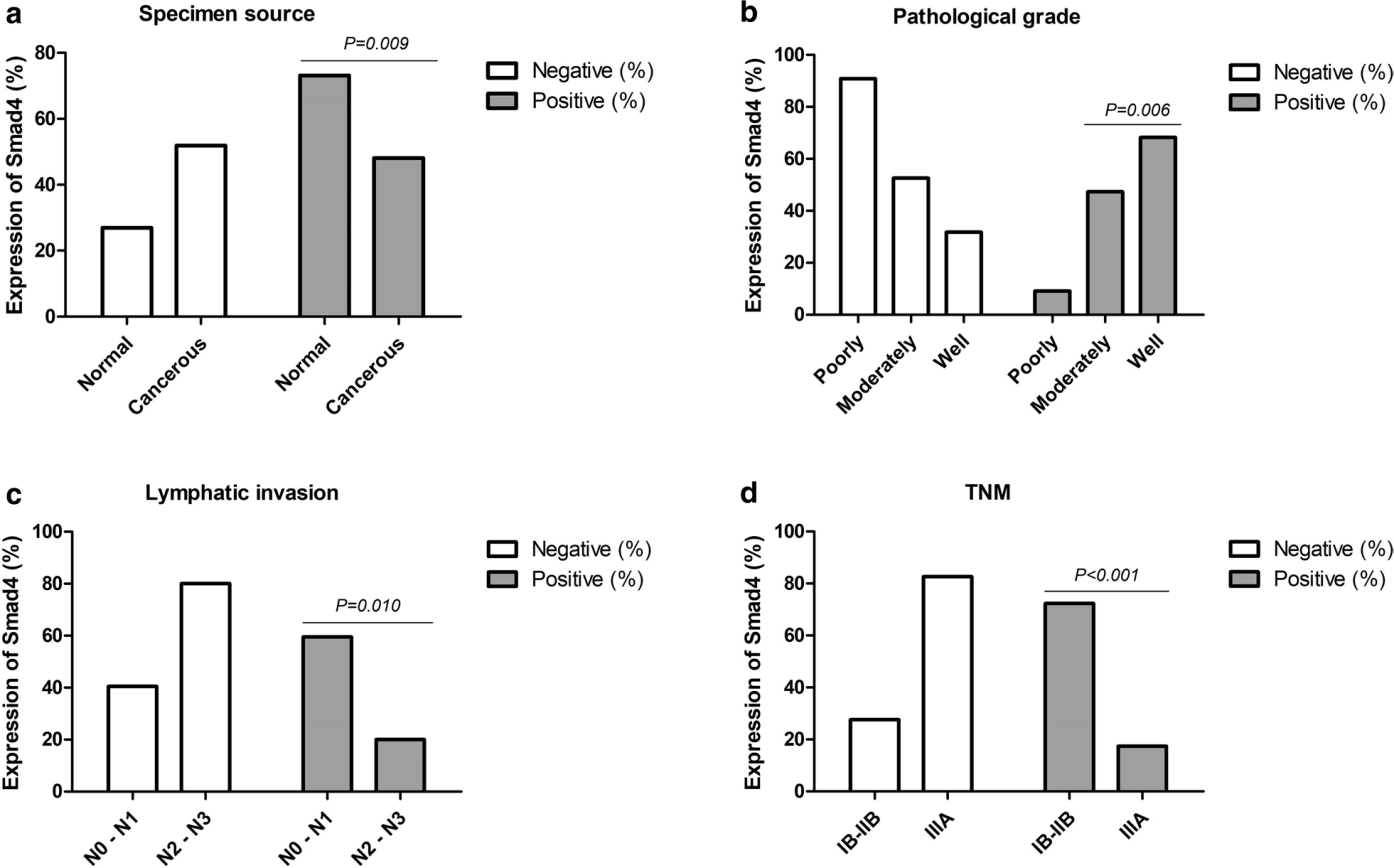
Correlation between clinical features and Smad4 expression in NSCLC tissues. **a** Expression of Smad4 was lower in NSCLC tissues than in normal lung tissues adjacent to cancer (*P* = 0.009). **b** Expression of Smad4 was lower in poorly differentiated NSCLC than in moderately and well differentiated NSCLC (*P* = 0.006). **c** NSCLC patients with lymph node metastasis (N2–N3) had a lower Smad4 expression than patients with lymph node metastasis (N0–N1) (*P* = 0.010). **f** NSCLC patients at stage IIIA showed a lower Smad4 expression than patients at stages IA–IIB (*P* < 0.001). NSCLC, non-small cell lung cancer; LAC, lung adenocarcinoma. LSCC, lung squamous cell carcinoma. N, node stage (TNM classification)

Immunohistochemistry was used to detect the expression of Smad4 in lung cancer tissues and normal cancer-adjacent lung tissues. The positive signal was stained brown-yellow, most of which was expressed in the cytoplasm (Fig. 5a–f). The results showed that the positive expression rate of Smad4 in NSCLC tissues was 48.1% (25/52), while the positive expression rate in normal cancer-adjacent lung tissues was 73.1% (38/52) (Table 6; Fig. 6a). The results indicates that the expression of Smad4 in NSCLC is lower than that in normal tissues (*P* = 0.009).

**Table 6:** Expression of Smad4 in lung cancer tissues (N = 52)

Parameter	Group	N	Expression of Smad4			
			Negative (%)	Positive (%)	χ^2^ value	*P* value
Specimen source	Normal	52	14 (26.9)	38 (73.1)	5.798	0.009
	Cancerous	52	27 (51.9)	25 (48.1)		
Gender	Male	31	15 (48.4)	16 (51.6)	0.384	0.535
	Female	21	12 (57.1)	9 (42.9)		
Ages	< 60	29	15 (51.7)	14 (48.3)	0.001	0.974
	≥ 60	23	12 (52.2)	11 (47.8)		
Smoking	No	27	13 (48.1)	14 (51.9)	0.321	0.571
	Yes	25	14 (56)	11 (44)		
Histology	LAC	19	12 (63.2)	7 (36.8)	1.514	0.219
	LSCC	33	15 (45.5)	18 (54.5)		
Pathological grade	Poorly	11	10 (90.9)	1 (9.1)	10.264	0.006
	Moderately	19	10 (52.6)	9 (47.4)		
	Well	22	7 (31.8)	15 (68.2)*		
Lymph node metastasis	N0–N1	37	15 (40.5)	22 (59.5)	6.657	0.010
	N2–N3	15	12 (80)	3 (20)		
TNM	IB-IIB	29	8 (27.6)	21 (72.4)	15.55	< 0.001
	IIIA	23	19 (82.6)	4 (17.4)		

*N* numbers of patients, *LAC* lung adenocarcinoma, *LSCC* lung squamous cell carcinoma, *TNM* the TNM Classification of Malignant Tumours

*p < 0.05, well differentiated tissues compared with poorly differentiated tissues

### Expression of Smad4 in NSCLC tissues is related to the degree of tissue differentiation, lymph node metastasis and clinical stage

Compared with the well differentiated tissues (68.2%; 15/22), the expression of Smad4 was downregulated in poorly differentiated NSCLC tissues (9.1%; 1/11) (*P* = 0.006) (Table 6; Fig. 6b). And the expression of Smad4 in NSCLC tissues with N2–N3 stage (20%; 3/15) of lymph node metastasis was lower than in NSCLC tissues with N0–N1 (59.5%; 22/37) stage (*P* = 0.010) (Table 6; Fig. 6c). The expression of Smad4 in NSCLC tissues with clinical stage IIIA (17.4%; 4/23) was lower than that in NSCLC tissues with clinical stage IB–IIB (72.4%; 21/29) (*P* < 0.001) (Table 6; Fig. 6d).

### Expression level of Smad4 in serum and cancer tissues of NSCLC patients has a consistent trend

The cut-off value of serum Smad4 at 122 ng/L was used as a delimitation, the value greater than this value was defined as positive, and the value less than or equal to this value was defined as negative. This statistical result showed that the positive co-expression rate of Smad4 in serum and NSCLC tissues was 48.08% (25/52), and the negative co-expression rate was 40.41% (21/52). The Kappa value was 0.771, and the asymptotic standard error was 0.086; the former was significantly larger than the latter (Kappa=0.771>0.086), which suggests that the two inspection methods are in good agreement (*T*=5.771, *P*<0.001).

## Discussion

The purpose of our study was to analyze the relationship between Smad4 and NSCLC and accumulate clinical data for molecular diagnosis and treatment based on Smad4. We summarized the findings as follows. The expression level of Smad4 in the serum and lung cancer tissues of patients with lung cancer was lower than that in the non-tumor group. Histological analysis showed that the expression of Smad4 in adenocarcinoma was lower than that in squamous cell carcinoma. On the level of tumor burden analysis, the level of Smad4 in patients with higher-grade stages was lower than that of patients with lower-grade stages. In addition, the expression level of Smad4 was decreased in patients with lymph node metastasis and later stage. The results suggest that the downregulation or deletion of Smad4 is related to the malignant biological behavior of NSCLC and serum Smad4 could be considered as a potential molecular indicator for diagnosis and evaluation of NSCLC. The Smad4 protein encoded by the DPC4 gene belongs to the Smad family and can be activated by transmembrane serine/threonine receptor kinases, such as transforming growth factor-β (TGF-β) receptors, and thus serves as an important intracytoplasmic signaling cascade molecule for TGF-β signaling. Smad4 can form homologous complexes by itself or heterologous complexes with other activated Smad family members, transfer to the nucleus, and cooperate with other transcription factors to regulate the transcription of TGF-β response genes. Inactivation or low expression of Smad4 may affect TGF-β signal transduction and participate in tumor formation [11, 12]. Our findings that serum concentration of Smad4 in NSCLC patients was higher than that in patients with benign lung disease and healthy individuals means that the downregulation of Smad4 is linked to the occurrence and development of NSCLC. A previous study showed that the expression of Smad4 is absent in pancreatic cancer, and its deletion can promote the progress of pancreatic cancer and increase the tumor metastasis [13]. And the loss of Smad4 expression is also found in squamous cell carcinoma of the head and neck and esophageal squamous cell carcinoma, and the low expression of Smad4 is closely related to the aggressive behavior of these two tumors [14, 15]. Our research results are consistent with the conclusions of the above studies.

Through subgroup analysis, we found that the serum Smad4 concentration of LAC patients was lower than that of LSCC patients. This finding not only indicates that the downregulation or deletion of Smad4 is related to the occurrence and development of LAC, but also implies that the serum concentration of Smad4 is also conducive to the identification of LAC and LSCC. A previous study for cancer genome map research shows that 13% of LSCC and 47% of LAC have Smad4 deletion [16]. The deletion of Smad4 can promote the formation of lung cancer by inhibiting DNA repair and at the same time make the tumor itself more sensitive to topoisomerase inhibitors, which is related to the regulatory mechanism of DNA repair of Smad4 [17]. Although we observed that the serum Smad4 concentration of female patients was lower than that of males, we found that it was related to the high rate of LAC in female patients through data analysis. In pathology, NSCLC is divided into high-differentiation, middle-differentiation and low-differentiation according to the degree of cancer cell differentiation. The speed of progression and metastasis in NSCLC have been confirmed to be related to the degree of differentiation, that is, the lower the differentiation, the higher the degree of malignancy. In our study, we found that the serum concentration of Smad4 in poorly differentiated NSCLC was in turn lower than that of moderately differentiated and well differentiated NSCLC, which implies that Smad4 plays a potential role in the disease progression of NSCLC. Recently, it is believed that the loss of Smad4 itself may not directly initiate tumor formation, but it can promote tumor progression initiated by other genes, such as KRAS activation in pancreatic ductal adenocarcinoma and APC inactivation in colorectal cancer, indicating that Smad4 plays different roles in different tumors [8]. We also found that the serum concentration of Smad4 was negatively correlated with the degree of lymph node metastasis and that the serum Smad4 in patients with late clinical stage was lower than that in patients with early clinical stage. A previous study shows that the expression of Smad4 in gastric cancer tissue is significantly lower than that in normal tissues adjacent to the cancer, especially in poorly differentiated tissues; and the expression of Smad4 in patients with lymph node metastasis is lower than in those without lymph node metastasis [18]. And some studies have reported that Smad4 is an important transcriptional regulator of TGF-β pathway and the loss of Smad4 function is related to the occurrence and evolution of a variety of solid tumors, especially related to the metastasis of pancreatic cancer, ovarian cancer, breast cancer, and intestinal cancer [19–22]. A study has confirmed that Smad4 can regulate the metastasis process of lung cancer by inhibiting the expression of VEGF [23]. Combined with our research and existing reports, we believe that the reduction of serum Smad4 concentration is related to the occurrence and development of NSCLC and Smad4 may be a potential target for clinical diagnosis and treatment of NSCLC.

In order to verify the ability of serum Smad4 concentration to screen NSCLC, we conducted a ROC curve analysis. We found that the sensitivity and specificity of serum Smad4 concentration to distinguish NSCLC from healthy individuals were 91.56% and 61.56% while the sensitivity and specificity of serum Smad4 concentration to distinguish NSCLC from benign lung disease were 84.55% and 60.36%. We converted the Smad4 serum concentration into binary variables for Logistic regression analysis and found that the degree of cell differentiation, lymph node metastasis and NSCLC clinical stage directly affected the expression level of Smad4, which was significantly negatively correlated with the expression of Smad4. The results indicate that the serum concentration of Smad4 can be used to evaluate the degree of malignant biology of NSCLC. Because it is directly related to the metastasis and clinical progress of NSCLC, it can be used as a prognostic index. There are many target genes regulated by SMAD4, and the transcription factor effect of SMAD4 can directly regulate the target genes, thereby increasing the risk of cell canceration [6, 8, 10, 13, 16, 21]. At present, the research on Smad4 mainly focuses on the prognosis evaluation and metastasis evaluation of tumors. However, the enhancement or restoration of Smad4 expression may regulate the signal transduction pathways involved, such as TGF-β/smad4, etc., and may provide new strategies for tumor treatment [8, 10, 16]. We further found that the expression of Smad4 in NSCLC was lower than that in normal lung tissue adjacent to cancer and that Smad4 was downregulated or deleted in poorly differentiated, lymph node metastatic, and later clinical stage NSCLC. The loss of Smad4 expression is more common in pancreatic, bile duct, appendix, and colon tumors. It also has a certain degree of loss in breast cancer, esophagus, and gastric adenocarcinoma, and the loss is often accompanied by a positive CK7 [24]. Our study explored the changes of Smad4 in NSCLC and showed that its change could be used as a potential indicator for evaluating the degree of NSCLC differentiation and lymph node metastasis. However, how it participates in the signal transduction process of cells during the occurrence of NSCLC and which signaling pathways participate in the proliferation and metastasis of NSCLC remains to be further studied. There are several unsatisfactory points in this study. We summarize them as follows: 1) this study did not discuss SMAD4 at the genetic level, such as the mutation of Smad4 in NSCLC; 2) this study did not involve the study of Smad4 signaling mechanism in NSCLC; and 3) the population in this study was all Chinese, which may lead to a risk of regional and ethnic deviation.

## Conclusions

The expression level of Smad4 in the serum and cancer tissues of NSCLC patients is decreased and the decreased Smad4 is related to poorly differentiated cancer, lymphatic metastasis and advanced TNM staging. The change of serum Smad4 is helpful for screening and evaluation of NSCLC. This results indicates that the downregulation or deletion of Smad4 is related to the malignant biological behavior of NSCLC, and in-depth study of Smad4 may bring new ideas for the diagnosis and treatment of NSCLC.

## Data Availability

The datasets supporting the conclusions of this article are included within the article. The datasets used and/or analyzed during the current study are available from the corresponding author on reasonable request.

## Abbreviations

APC: Adenomatous polyposis coli
APES: 3-Aminopropyl triethoxysilane
AUC: Area under the ROC curve
CK7: Cytokeratin 7
CT: Computed tomography
DAB: 3,3′-Diaminobenzidine tetrahydrochloride
DNA: Deoxyribonucleic acid
DPC4: Deleted in pancreatic carcinoma locus 4
ELISA: Enzyme-linked immunosorbent assay
HRP: Horseradish peroxidase
IHC: Immunohistochemistry
KRAS: V-Ki-ras2 Kirsten ratsarcoma viral oncogene homolog
LAC: Lung adenocarcinoma
LSCC: Lung squamous cell carcinoma
NSCLC: Non-small cell lung carcinomas
ROC: Receiver operating characteristic
SE: Standard error
TGF-β: Transforming growth factor-β
VEGF: Vascular endothelial grown factor
